# COVID-19 and Congenital Cleft Lip and Palate: A Systematic Review Focusing on Nonsyndromic Neonatal Outcomes

**DOI:** 10.7759/cureus.106878

**Published:** 2026-04-12

**Authors:** Praisy Joy, Roopa Kunthavai, Suchanda Sahu, Samapika Routray

**Affiliations:** 1 Anatomy, All India Institute of Medical Sciences, Bhubaneswar, Bhubaneswar, IND; 2 Orthodontics and Dentofacial Orthopedics, Government Dental College, Cuddalore, Cuddalore, IND; 3 Biochemistry, All India Institute of Medical Sciences, Bhubaneswar, Bhubaneswar, IND; 4 Dentistry, All India Institute of Medical Sciences, Bhubaneswar, Bhubaneswar, IND

**Keywords:** covid-19, developmental disturbances, maternal, neonates, pandemic

## Abstract

Craniofacial anomalies (CFAs), such as cleft lip and palate, are among the most frequent birth defects, often necessitating multidisciplinary care. The COVID-19 pandemic raised concerns that maternal SARS-CoV-2 infection and associated factors, such as fever, inflammation, and psychosocial stress, might elevate teratogenic risk. This systematic review aims to synthesize the evidence on the association between maternal COVID-19 exposure and nonsyndromic CFAs in neonates. We conducted a systematic review in accordance with the PRISMA 2020 guidelines. Major electronic databases (PubMed/MEDLINE, Embase, Web of Science, Cochrane Library, and the WHO COVID-19 Database) were searched from their inception until February 2025. We included studies examining the association between documented maternal COVID-19 infection (before or during pregnancy) and the incidence, type, or severity of CFAs, with a focus on nonsyndromic orofacial clefts (NSOFC). Risk of bias of the studies was assessed using the ROBINS-I tool. Findings were synthesized narratively, and bibliometric analyses, including geo-mapping and conceptual structure mapping, were performed. Eleven studies met the inclusion criteria. The evidence was mixed: smaller regional studies suggested a possible link, particularly with first-trimester infection, which aligns with the critical timing of craniofacial development. However, large-scale, robust epidemiological and registry-based studies from the USA and Nordic countries consistently found no significant association between maternal COVID-19 infection and congenital anomalies. A meta-analysis could not be performed due to study heterogeneity. Maternal stress and fear of COVID-19 were repeatedly highlighted as potential factors, in some cases showing stronger or even paradoxical associations than the infection itself. Research activity was concentrated primarily in Saudi Arabia and the United States. Robust population-level evidence does not currently support a direct, major causal link between maternal SARS-CoV-2 infection and NSOFC. While a modest or context-specific association cannot be entirely excluded, the effects of maternal psychosocial stressors and restricted healthcare access during the pandemic appear to be important explanatory factors that warrant further investigation, independent of the viral infection itself.

## Introduction and background

Craniofacial anomalies (CFAs) are a heterogeneous group of congenital structural and functional disorders of the skull and face, varying widely in severity. They may be classified by etiology (genetic, environmental, or multifactorial), anatomy (cranial, orbital, nasal, oral, mandibular/maxillary, or auricular), syndromic association (syndromic vs nonsyndromic), or pathogenesis (malformations, deformations, disruptions, or dysplasias). Common examples include cleft lip and palate (CL/P), craniosynostosis, micrognathia, microtia, and choanal atresia. Globally, CFAs are among the most frequent birth defects, with CL/P affecting approximately 1 in 700 live births [1]. They often impair feeding, speech, hearing, and psychosocial well-being, necessitating multidisciplinary care.

The COVID-19 pandemic added challenges to CFA management and research. Maternal infection, hypoxia, fever, inflammation, and psychosocial stress may elevate teratogenic risk, while restricted access to prenatal care, delayed surgeries, and disrupted therapies adversely impact outcomes. Thus, understanding the intersection of CFAs and COVID-19 is vital for improving counseling, care, and long-term prognosis.

Craniofacial morphogenesis primarily occurs during the first trimester, between the fourth and 12th weeks of gestation. It begins with the migration and proliferation of neural crest cells, which form the facial prominences. By the fifth to seventh weeks, the frontonasal, maxillary, and mandibular processes develop and fuse to shape the upper lip and primary palate. Palatogenesis continues from the seventh to 12th weeks, when the secondary palate forms through elevation and fusion of the palatal shelves. Disruptions during this critical window, due to genetic or environmental factors, can result in anomalies such as cleft lip and palate.

Pathogenesis of CFAs

CFAs originate during early embryogenesis, often due to genetic mutations, chromosomal abnormalities, or environmental insults such as teratogens, infections, or nutritional deficiencies. Neural crest cell dysfunction, affecting migration, proliferation, or differentiation, is central to many anomalies. Syndromic cases (e.g., Apert and Treacher Collins) have defined genetic bases, whereas nonsyndromic cases often involve complex signaling disruptions (e.g., TGF-β, SHH, and FGFR pathways).

The mechanistic (or mammalian) target of rapamycin (mTOR) is a serine/threonine protein kinase present universally in all eukaryotic cells. It is a central cellular signaling pathway that controls how cells grow, divide, and respond to their environment. It integrates signals from nutrients, growth factors, oxygen, and energy status to regulate essential processes such as protein synthesis, metabolism, and cell survival. The mTOR functions as part of two complexes: mTORC1, which regulates protein synthesis, cell growth, lipid and nucleotide synthesis, and inhibits autophagy; and mTORC2, which controls cell survival, cytoskeleton organization, and metabolism. Dysregulation, such as overactivation, is linked to cancer or metabolic disorders [2], whereas repression leads to neurodevelopmental syndromes [3] and CFAs [4].

The mTOR pathway is pivotal in craniofacial development, regulating cranial neural crest cell proliferation, migration, differentiation, and survival. Dysregulation can cause undergrowth (e.g., cleft palate) or overgrowth (facial asymmetries and craniosynostosis). Mutations in mTOR regulators (TSC1, TSC2, PTEN, and PIK3CA) are associated with syndromes such as tuberous sclerosis [5] and Cowden syndrome. Mouse and zebrafish models have demonstrated that the level of protein in the maternal diet modulates mTORC1 activity during embryogenesis. Both insufficient and excessive mTORC1 activity led to altered facial morphologies, demonstrating the critical importance of fine control of mTORC1 in normal craniofacial development [6].

**Figure 1:**
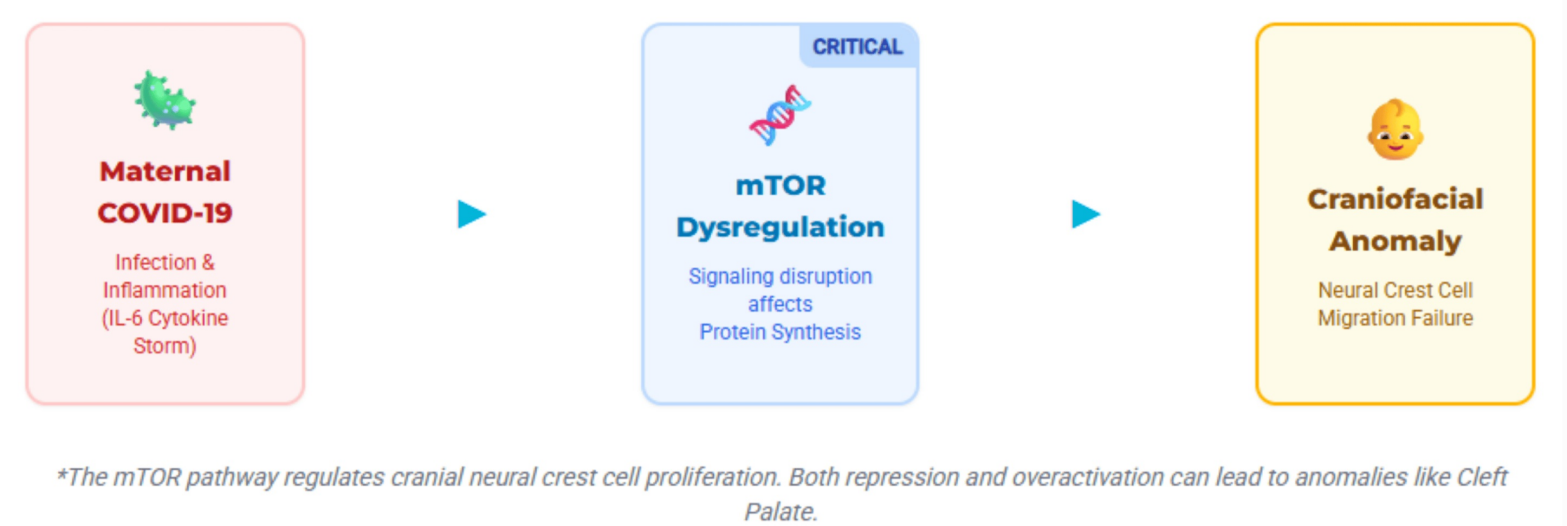
Proposed mechanistic pathway linking maternal COVID-19 infection to CFAs This schematic illustrates a proposed biological mechanism by which maternal COVID-19 infection may contribute to the development of CFAs in the fetus. Maternal infection triggers systemic inflammation, including elevated cytokines such as IL-6, which may disrupt key developmental signaling pathways. Central to this mechanism is dysregulation of the mTOR pathway, a critical regulator of cell growth, proliferation, and protein synthesis. Altered mTOR signaling can impair CNCC proliferation and migration, processes essential for normal craniofacial development. Disruption of these processes may result in anomalies such as cleft lip, cleft palate, and other craniofacial defects. Both hypoactivation and hyperactivation of the mTOR pathway can contribute to abnormal morphogenesis, highlighting its critical role in embryonic development. CFAs, craniofacial anomalies; CNCC, cranial neural crest cell; mTOR, mechanistic target of rapamycin This figure was prepared using Canva (Canva Pty Ltd., Sydney, Australia). Image credit: Roopa Kunthavai and Samapika Routray

In COVID-19, mTOR influences viral replication, immune modulation, and inflammation. SARS-CoV-2 exploits mTOR-driven protein synthesis, while dysregulated signaling contributes to lymphopenia and cytokine storms (notably IL-6-driven) (Figure 1) [7,8].

## Review

Methods

Eligibility Criteria

This review included studies that examined the association between maternal COVID-19 infection, either occurring before pregnancy or during gestation, and the development of CFAs in neonates. Eligible anomalies included cleft lip, cleft palate, craniosynostosis, and facial dysmorphia. The review was conducted in accordance with the PRISMA 2020 and PECO guidelines, as summarized in Table 1.

**Table 1:** PECO framework for the study This table presents the PECO framework used to define the research question and guide the systematic review. P (population) includes pregnant women and their offspring, focusing on in utero exposure. I (intervention/exposure) refers to maternal infection with COVID-19 during pregnancy. C (comparison) comprises noninfected pregnant women or pre-pandemic populations. O (outcome) includes CFAs, particularly orofacial clefts and related congenital malformations. This structured approach ensures clarity in study selection, data extraction, and synthesis of evidence regarding the association between maternal COVID-19 and craniofacial outcomes. CFAs, craniofacial anomalies

Component	Description
P (population)	Pregnant women and their offspring (fetuses/newborns), including those exposed in utero
I (intervention/exposure)	Maternal infection with COVID-19 (SARS-CoV-2) during pregnancy
C (comparison)	Pregnant women without COVID-19 infection or pre-pandemic populations
O (outcome)	Occurrence of CFAs, including orofacial clefts (cleft lip and cleft palate) and other congenital facial malformations

The population comprised neonates with confirmed exposure to SARS-CoV-2. Exposure was defined as documented maternal COVID-19 infection before or during pregnancy, while comparators included unexposed neonates where available. Outcomes of interest were the incidence, type, severity, and clinical characteristics of CFAs. Observational study designs, particularly cross-sectional studies, were included.

The time frame for inclusion was from December 2019 to February 2025. Studies published in English were considered, along with those in other languages, if reliable translations were available. Both peer-reviewed articles and gray literature, including preprints and registry data, were included.

Information Sources

A comprehensive literature search was conducted across multiple electronic databases, including PubMed/MEDLINE, Embase, Web of Science, the Cochrane Library, and the WHO COVID-19 Global Literature Database, from their inception until February 2025. Additional sources included manual screening of reference lists from included studies, clinical trial registries, and communication with experts in the field to identify unpublished or ongoing studies. This multisource approach was adopted to ensure a thorough and unbiased capture of relevant literature.

Search Strategy

The search strategy was developed in collaboration with an information specialist from the institutional library to ensure methodological rigor and comprehensiveness. It combined keywords related to COVID-19 (e.g., “COVID-19”, “SARS-CoV-2”, “novel coronavirus”) with terms related to craniofacial anomalies (e.g., “craniofacial anomalies”, “cleft lip”, “cleft palate”, “facial dysmorphia”). Controlled vocabulary, such as MeSH terms in PubMed and Emtree terms in Embase, was applied where appropriate. Boolean operators were used to refine the search, and strategies were tailored for each database. Search limits regarding publication date and language were aligned with the predefined eligibility criteria. All strategies were peer-reviewed or independently verified to ensure accuracy and sensitivity.

Search String 

The search string used for PubMed was as follows: ("COVID-19"[Mesh] OR "SARS-CoV-2"[Mesh] OR covid-19 OR covid OR coronavirus OR "2019-nCoV" OR "SARS-CoV-2") AND (pregnan* OR maternal OR fetus OR fetal OR prenatal OR antenatal) AND ("Craniofacial Abnormalities"[Mesh] OR "Cleft Lip"[Mesh] OR "Cleft Palate"[Mesh] OR craniofacial anomal* OR orofacial cleft* OR cleft lip OR cleft palate OR facial malformation* OR congenital anomal*).

The search string used for Embase was as follows: ('covid-19'/exp OR 'sars cov 2'/exp OR covid OR coronavirus) AND (pregnancy/exp OR maternal OR fetal OR prenatal OR antenatal) AND ('craniofacial malformation'/exp OR 'cleft lip'/exp OR 'cleft palate'/exp OR craniofacial OR orofacial cleft OR congenital anomaly).

The search string used for Google Scholar was as follows: ("COVID-19" OR "SARS-CoV-2" OR coronavirus) AND (pregnancy OR maternal OR prenatal OR antenatal OR fetal) AND ("craniofacial anomalies" OR "orofacial clefts" OR "cleft lip" OR "cleft palate" OR "facial malformations" OR "congenital anomalies").

Study Selection

**Figure 2:**
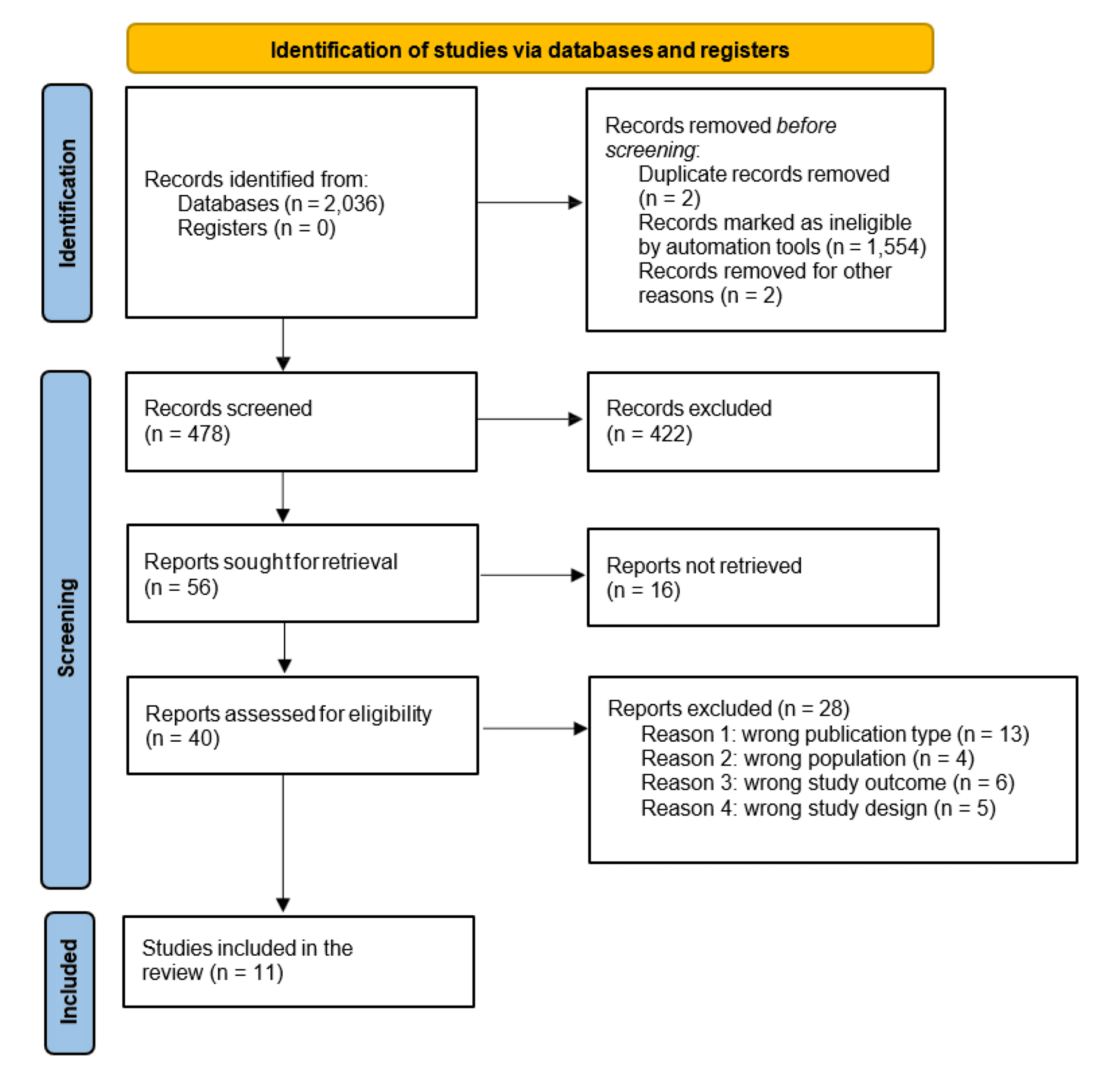
PRISMA 2020 flow diagram of study selection process This figure illustrates the study selection process following PRISMA 2020 guidelines. A total of 2,036 records were identified through database searching, with no records from registers. After removal of duplicates, automated exclusions, and irrelevant records, 478 studies were screened. Of these, 422 were excluded based on title and abstract screening. Fifty-six reports were sought for retrieval, with 16 not retrieved. Forty full-text articles were assessed for eligibility, and 28 were excluded for reasons such as wrong study design, population, outcome, or publication type. Ultimately, 11 studies were included in the systematic review.

All retrieved records were imported into the reference management software Rayyan (Rayyan Systems, Inc., Cambridge, MA, USA), where duplicates were identified and removed. Two independent reviewers conducted title and abstract screening based on the predefined eligibility criteria. Full-text articles of potentially relevant studies were subsequently assessed in duplicate. Any disagreements between reviewers were resolved through discussion, and when necessary, a third reviewer was consulted for arbitration. The entire selection process was documented and presented using a PRISMA 2020 flow diagram, detailing the number of records identified, screened, excluded, and included in the final analysis (Figure 2).

Data Collection Process

Data extraction was performed using a standardized and piloted data extraction form to ensure consistency and completeness. Two independent reviewers extracted data from each included study. Any discrepancies in extracted data were resolved through discussion or consultation with a third reviewer. When necessary, corresponding authors of the included studies were contacted to obtain missing or unclear information, thereby enhancing the accuracy and completeness of the dataset.

Data Items

The extracted data encompassed several domains. Bibliographic details included authors, year of publication, and journal. Study characteristics comprised study design, setting, and sample size. Population-related variables included maternal and neonatal demographics, as well as gestational age. Exposure-related information captured the timing and method of COVID-19 diagnosis. Outcome variables included the type, incidence, and severity of CFAs. Additional variables, such as follow-up duration, study location, funding sources, and declarations of conflicts of interest, were also recorded to provide contextual and methodological insights.

Risk of Bias Assessment

**Figure 3:**
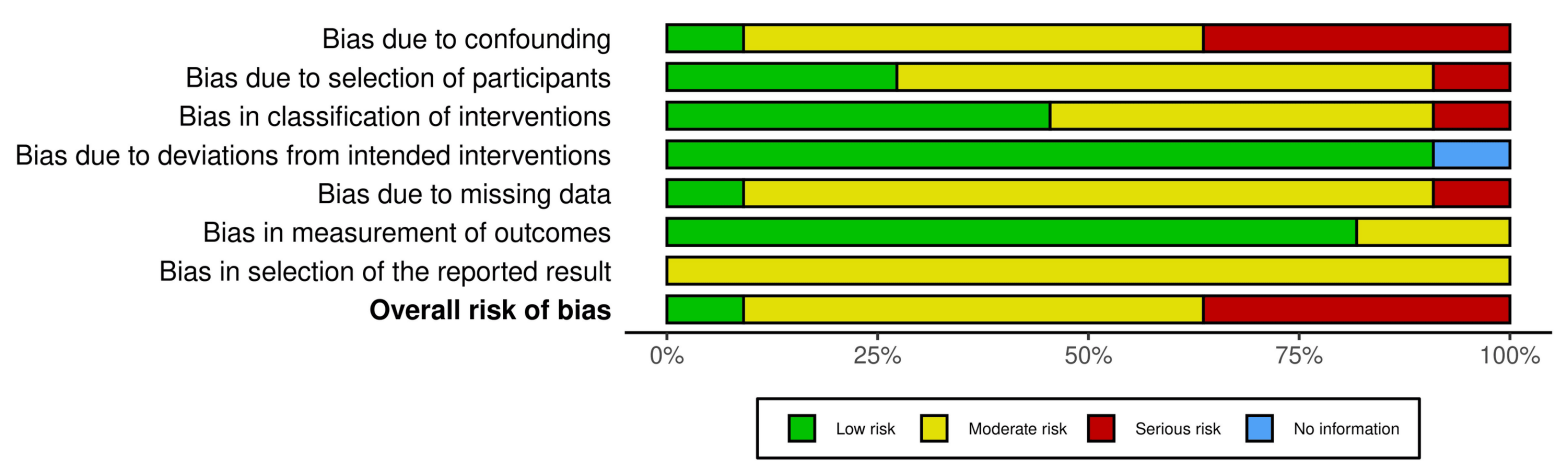
Risk of bias assessment of included studies using the ROBINS-I tool This figure summarizes the risk of bias across included studies using the ROBINS-I tool. Each bar represents the proportion of studies rated as low (green), moderate (yellow), serious (red), or no information (blue) across bias domains. Most studies demonstrated moderate to serious risk of bias, particularly due to confounding, participant selection, and missing data. Bias in the classification of interventions and outcome measurement was generally low. Selection of reported results was predominantly at moderate risk. Overall, the findings indicate methodological limitations across studies, suggesting that results should be interpreted with caution. ROBINS-I, Risk Of Bias In Non-randomized Studies of Interventions

**Figure 4:**
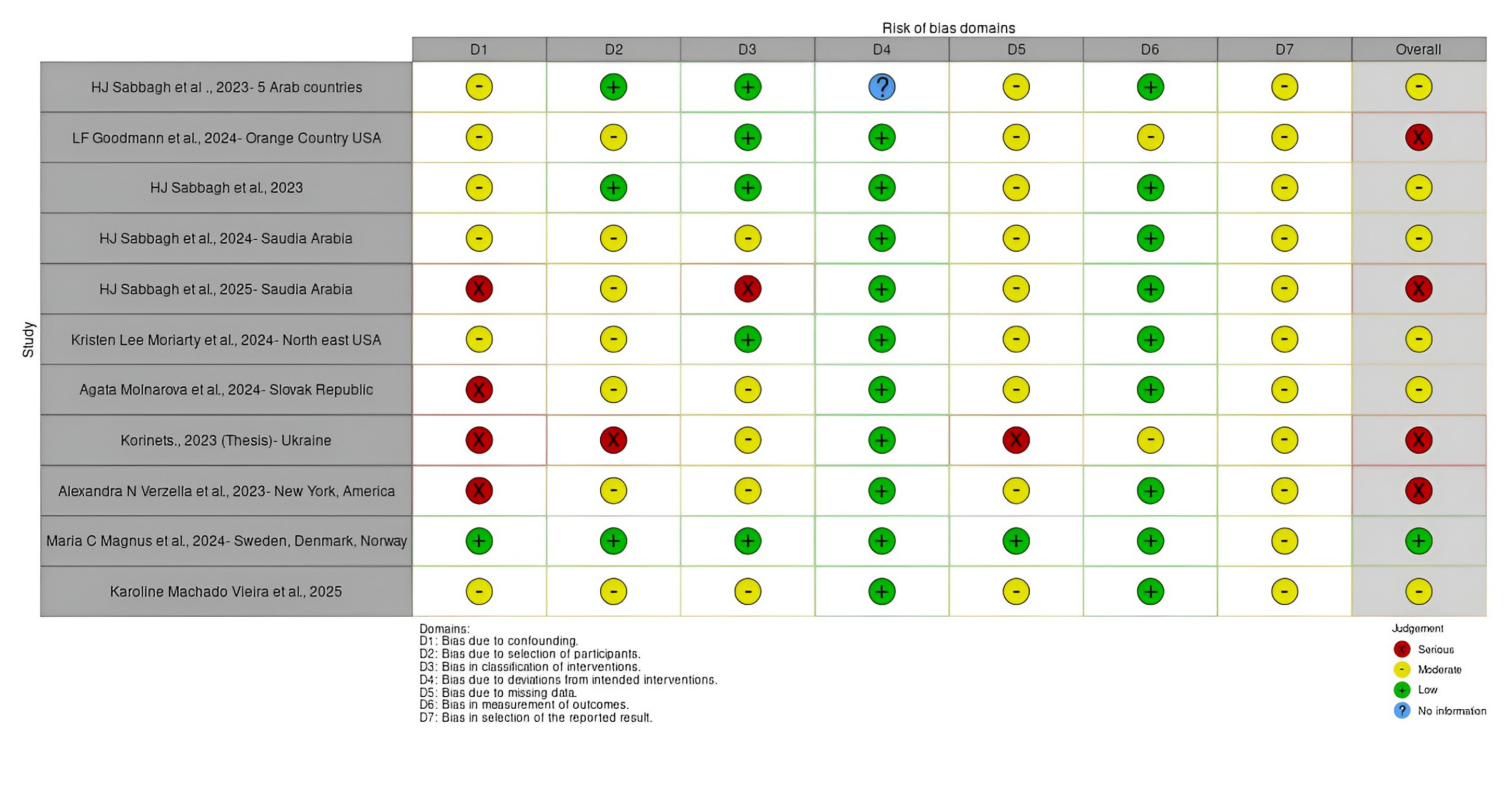
Risk of bias for individual studies using the ROBIN-I tool ROBINS-I, Risk Of Bias In Non-randomized Studies of Interventions

The risk of bias in included studies was independently assessed by two reviewers using the Risk Of Bias In Non-randomized Studies of Interventions (ROBINS-I) tool. This tool evaluates potential biases across multiple domains, including confounding, selection, classification of interventions, deviations from intended interventions, missing data, outcome measurement, and selective reporting. Any disagreements between reviewers were resolved through consensus. The resulting risk of bias assessments were incorporated into sensitivity analyses and were considered when interpreting the overall findings of the review (Figure 3). Figure 4 shows the risk of bias for individual studies.

Synthesis Methods

Data synthesis was primarily narrative, summarizing the findings across studies in a structured manner. Where sufficient homogeneous data were available, a quantitative synthesis was conducted using a random-effects meta-analysis model to account for between-study variability. Statistical heterogeneity was assessed using the I² statistic. These analyses aimed to explore potential sources of heterogeneity and provide more nuanced insights into the associations studied.

Statistical Analysis

All extracted data were initially organized and curated using Microsoft Excel 2021 (Microsoft Corporation, Redmond, WA, USA). A global distribution map of included studies was created using Datawrapper (Datawrapper GmbH, Berlin Germany) to visually represent their geographic origins. Bibliometric analyses, including hierarchical clustering dendrograms, keyword co-occurrence networks, and conceptual structure maps, were generated using RStudio (version 2025.05.1 Build 513; Posit PBC, Boston, MA, USA) with the Bibliometrix-Biblioshiny package and associated R libraries [9].

Results

Among the 11 studies selected for review, eight studies were against the null hypothesis, and 3 studies were in line with the null hypothesis. The details are given in Table 2. Across studies exploring the relationship between maternal COVID-19 exposure and nonsyndromic orofacial clefts (NSOFC) or CFAs, findings remain mixed.

**Table 2:** Details of included studies AOR, adjusted OR; CFAs, craniofacial anomalies; CL/CP, cleft lip and/or cleft palate; NSOFC, nonsyndromic orofacial cleft; SGA, small for gestational age

No.	Author and year	Study design	Population (N)	Intervention/exposure	Key outcomes	Findings
1	Sabbagh et al. (2023) [10] - five Arab countries	Case-control study, questionnaire-based	386 cases, 749 controls (1,135 infants from five Arab countries)	COVID-19 exposure	-	NSOFC may be associated with maternal lifetime exposure to stress and COVID-19 fear, with no direct effect of COVID-19.
2	Goodman et al. (2024) [11] - Orange County, USA	Retrospective cohort study	927,805 pediatric patients	COVID-19 exposure	OR - 2.87 (p < 0.001); eye, ear, face, neck - 1.16 (p < 0.001)	Severe COVID-19
3	Sabbagh et al. (2023) [12] - Saudi Arabia	Prospective study	177/140,380 - NSOFC	COVID-19	12.1% exposed; 13.2% vaccinated against COVID-19	National incidence of NSOFC in Saudi Arabia - 1.26 per 1,000 live births
4	Sabbagh et al. (2024) [13] - Saudi Arabia	Cross-sectional	273 infants with CL/CP	COVID-19	2.70 (p = 0.002) AOR for CL/CP	Mothers infected with COVID-19 during the first trimester had more than twofold higher odds of having an infant with a more severe CL/CP phenotype.
5	Sabbagh et al. (2025) [14] - Saudi Arabia	Retrospective cohort study	557 infants	COVID-19	0.43 (p = 0.008) AOR	The study shows a lower risk of NSOFC with fear of COVID-19 infection.
6	Moriarty et al. (2024) [15] - Northeast USA	Retrospective cohort study	Cases - 140; controls - 136	COVID-19	-	COVID-19 in the third trimester was associated with lower neonatal head circumference without SGA.
7	Molnárová et al. (2024) [16] - Slovak Republic	Prospective study	Cases - 13/24; total recruited - 2,729	COVID-19	Chi-square - 351.247, p < 0.01	Higher incidence of COVID-19 disease in mothers with an NSOFC child
8	Korinets and Prokopchuk (2023) [17] - Ukraine (thesis)	Cross-sectional, prenatal noninvasive USG study	159 pregnant women	COVID-19	Facial congenital anomalies - 5%	Facial anomalies were more prevalent and ranked first.
9	Verzella et al. (2023) [18] - New York, USA	Retrospective 10-year study, records from 173 institutes	163 million patient records	COVID-19	Craniosynostosis - 6.10; CL/CP - 10.22 and 2.02	CFAs are not associated with COVID-19.
10	Magnus et al. (2024) [19] - Sweden, Denmark, and Norway	Prospective Nordic registry-based study	343,066 live births	COVID-19	OR - 1.12 for CFA	COVID-19 during the first trimester was not associated with congenital anomalies.
11	Vieira et al. (2025) [20] - Brazil	Epidemiological time-series study	23,246/34,564,430; prevalence: 6.73 per 10,000 live births	COVID-19	6.73 per 10,000 live births	COVID-19 was not associated with the prevalence of orofacial clefts.

Smaller regional studies from Saudi Arabia, the Arab region, and Slovakia suggest a possible association, particularly when infection occurs during the first trimester, with some reporting increased severity of cleft phenotypes or higher incidence of NSOFC in exposed mothers. In contrast, large-scale epidemiological and registry-based studies from the USA, Nordic countries, and Brazil consistently found no significant association between COVID-19 infection and congenital anomalies, despite isolated signals in subgroup analyses.

Interestingly, maternal stress and fear of COVID-19 were repeatedly highlighted, in some cases showing stronger or even paradoxical associations than the infection itself, suggesting that psychological or contextual factors may influence outcomes. Overall, while early pregnancy infection has been flagged as a potential risk in smaller cohorts, robust population-level data do not support a direct causal link between COVID-19 and orofacial clefts.

**Figure 5:**
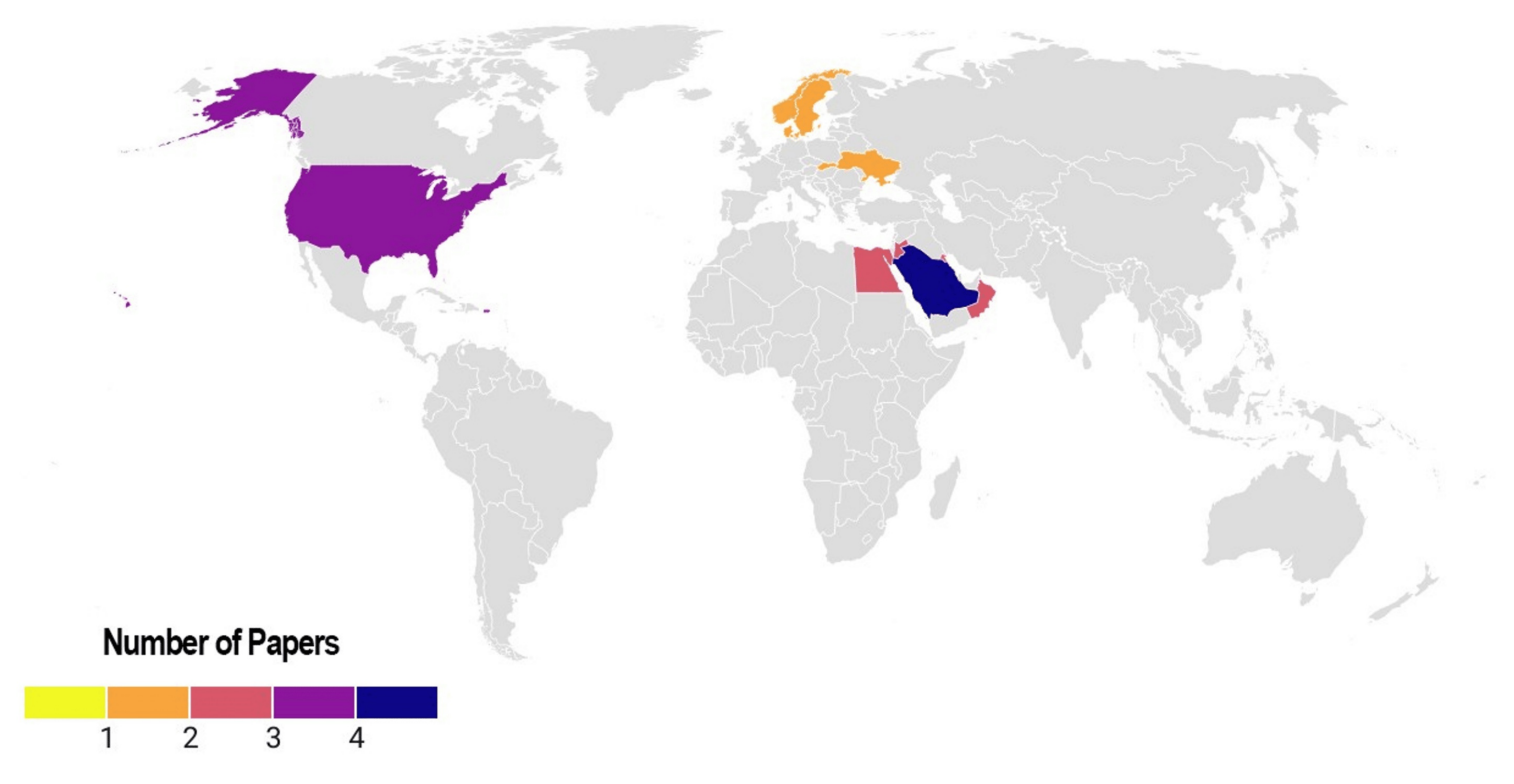
Geospatial map of the included studies This figure illustrates the global distribution of studies included in the systematic review. Countries contributing to the evidence base are highlighted on the world map, demonstrating the geographic spread of research on maternal COVID-19 and CFAs. The map reveals that most studies originated from specific regions, with limited representation from low- and middle-income countries. Variations in geographic distribution may reflect differences in research capacity, reporting systems, and pandemic burden. This visualization highlights potential gaps in global evidence and underscores the need for more geographically diverse studies to improve the generalizability of findings. CFAs, craniofacial anomalies This figure was created using RStudio. Image credit: Samapika Routray

The statistical geo-mapping analysis revealed that most studies were conducted in Saudi Arabia (n = 4) and the United States (n = 3), followed by smaller contributions from Egypt, Jordan, Kuwait, Oman, Slovakia, Sweden, and Ukraine (n = 1-2 each). This indicates that research activity has been concentrated primarily in the Middle East and North America, with scattered contributions from European countries (Figure 5).

**Figure 6:**
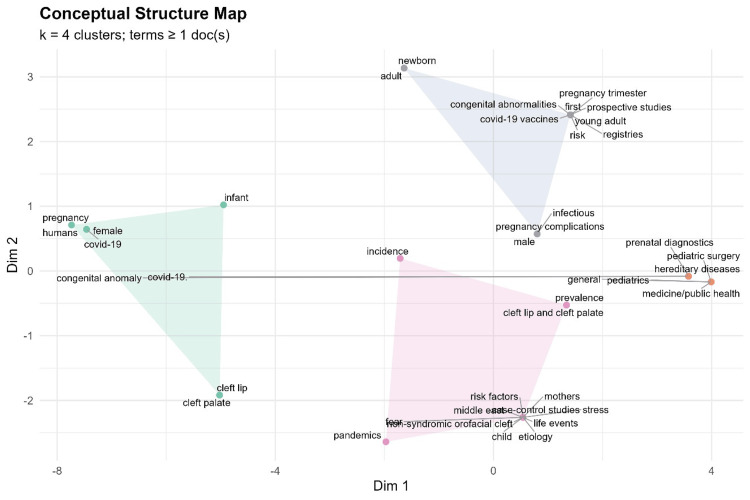
Conceptual structural map of the included studies This figure presents the conceptual structure map derived from bibliometric analysis, illustrating the thematic organization of the included studies. The map is based on keyword co-occurrence and multiple correspondence analysis, grouping related terms into distinct conceptual clusters. Each point represents a keyword, and proximity between points indicates conceptual similarity and co-occurrence within the literature. Clusters reflect major research themes, such as maternal COVID-19 exposure, CFAs, developmental mechanisms, and clinical outcomes. The relative positioning of clusters highlights relationships between themes, while their separation indicates conceptual distinctions, providing an overview of the intellectual structure and research focus within the field. CFAs, craniofacial anomalies This figure was created using RStudio. Image credit: Samapika Routray

To explore the thematic organization of research, a conceptual structure map based on multidimensional scaling and k-means clustering was constructed (Figure 6). The map highlighted four thematic clusters: one linking pregnancy, COVID-19, humans, females, and infants; a second centered on congenital anomalies, cleft lip and palate, and incidence; a third grouping terms such as pandemics, case-control studies, risk factors, and stress; and a smaller, distinct cluster including adults, newborns, prospective studies, and registries. This conceptual mapping suggests that the literature is structured around maternal-fetal health outcomes, congenital anomalies, and the broader effects of the pandemic on pregnancy.

**Figure 7:**
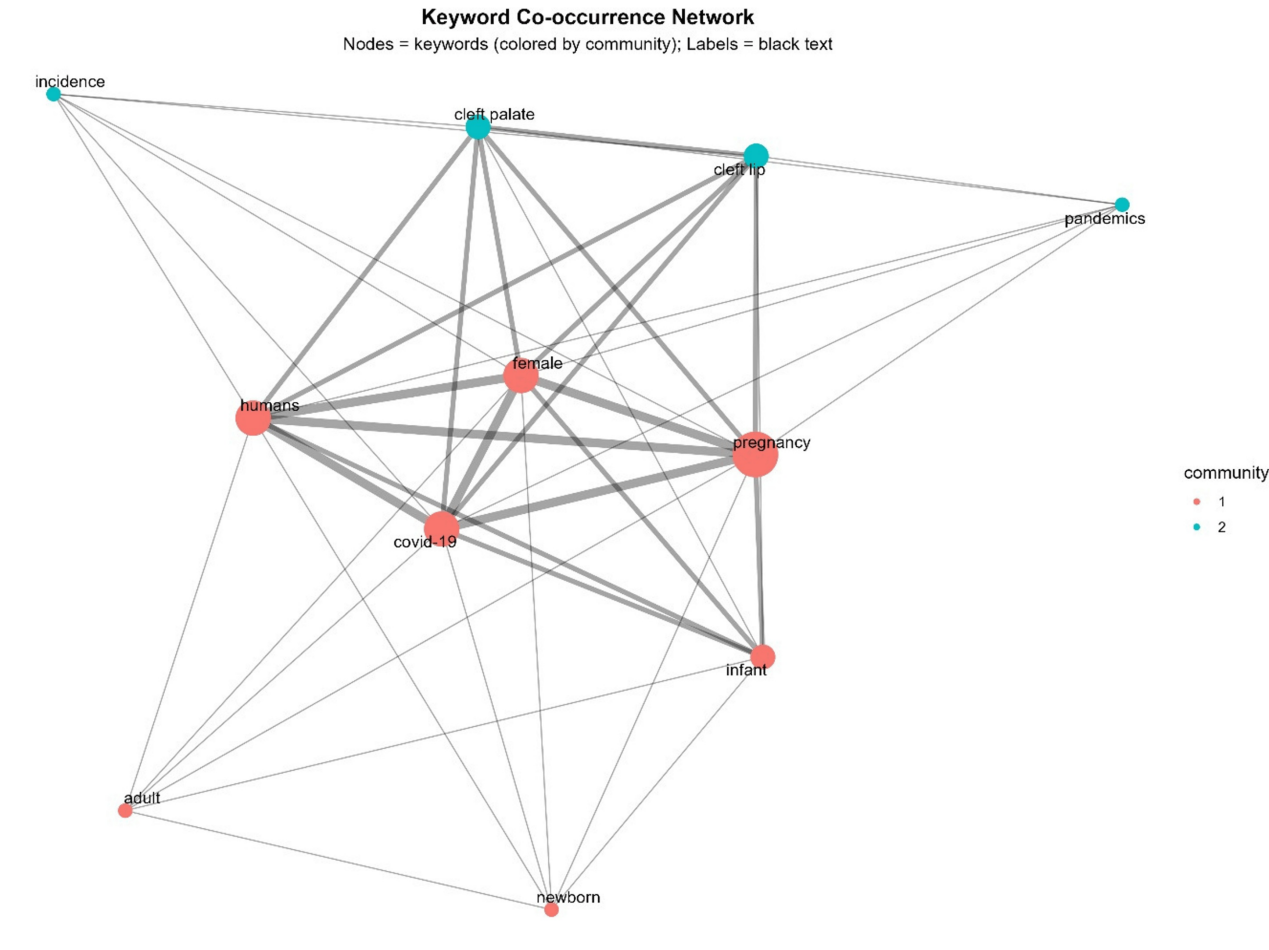
Keyword co-occurrence network This figure illustrates the keyword co-occurrence network generated from the included studies, highlighting relationships between frequently used terms in the literature. Each node represents a keyword, with node size proportional to its frequency of occurrence. Links between nodes indicate co-occurrence within the same study, with thicker lines representing stronger associations. Clusters of closely connected nodes reflect major thematic areas, such as COVID-19 infection, pregnancy, CFAs, and molecular pathways. Different colors denote distinct clusters identified through network analysis. This visualization provides insights into research trends, thematic structures, and the intellectual organization of the field. This figure was created using RStudio. Image credit: Samapika Routray

A keyword co-occurrence network further emphasized the prominence of pregnancy, COVID-19, humans, and females, which emerged as the largest and most connected nodes. These keywords were strongly associated with infant and congenital anomalies, while more peripheral associations were observed with pandemics and incidence (Figure 7).

**Figure 8:**
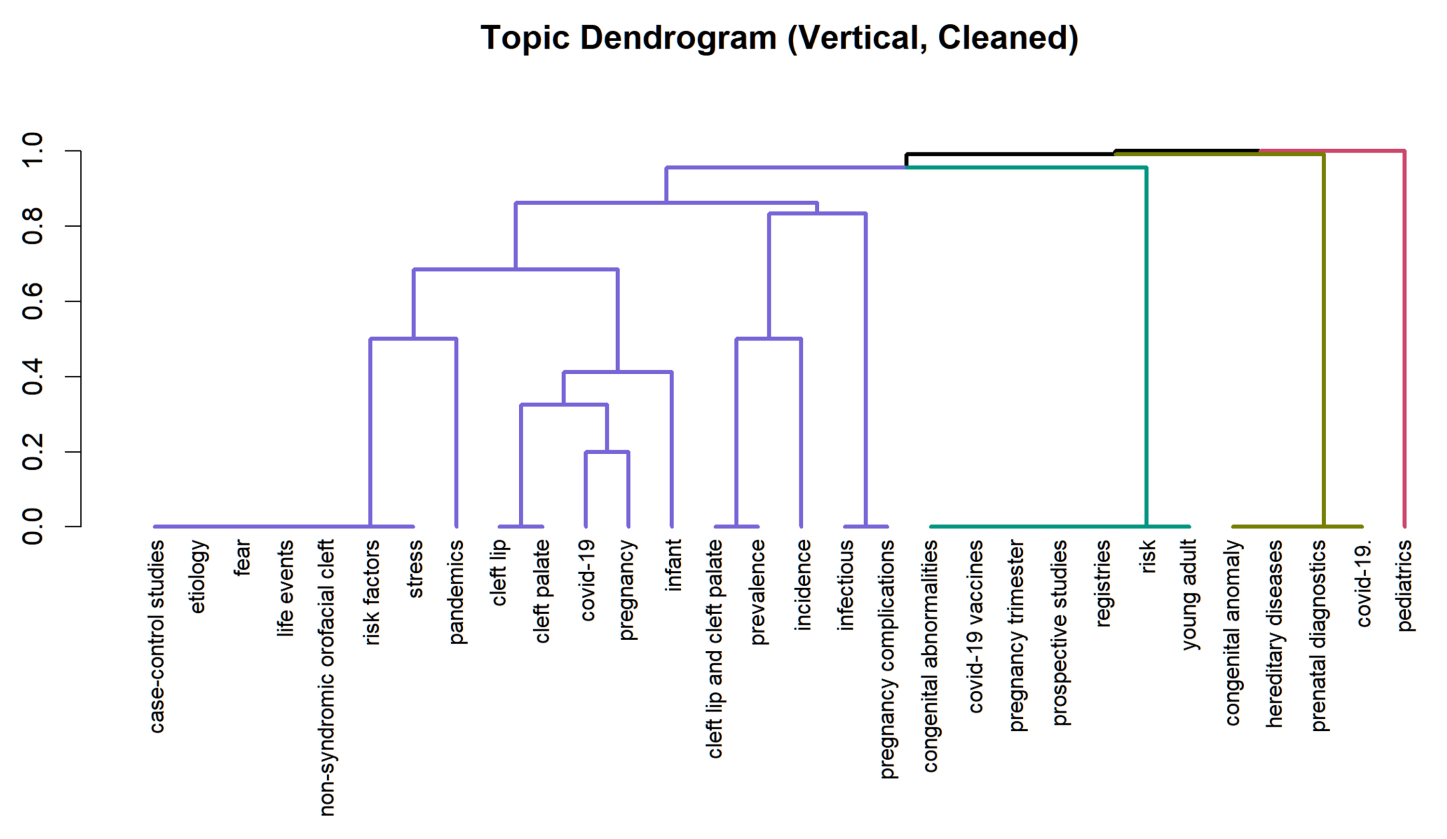
Hierarchical clustering dendrogram of included studies/keywords This figure presents a hierarchical clustering dendrogram illustrating the relationships among included studies (or keywords) based on similarity measures derived from bibliometric analysis. The dendrogram groups items into clusters according to shared characteristics, such as co-occurrence patterns or thematic similarity. The length of the branches reflects the degree of dissimilarity between clusters, with shorter distances indicating closer relationships. Distinct clusters represent major research themes within the literature, such as COVID-19 exposure, CFAs, and underlying molecular mechanisms. This visualization aids in identifying dominant topics, research trends, and conceptual structures within the included studies. CFAs, craniofacial anomalies This figure was created using RStudio. Image credit: Samapika Routray

A hierarchical clustering dendrogram was generated to visualize the similarity structure among keywords. Four main branches were evident, separating maternal and neonatal descriptors such as pregnancy, infant, and newborn from congenital anomaly-related terms, including cleft lip, cleft palate, and orofacial clefts. Other branches grouped methodological descriptors, such as prospective studies and registries, with broader etiological terms, including pandemics, stress, and risk factors (Figure 8).

A meta-analysis could not be performed as planned because incidence before and after the pandemic could not be compared across the 11 included studies. The pooled risk could not be calculated due to the heterogeneity of the studies.

Discussion

The current body of evidence examining COVID-19 and orofacial clefts presents a complex and somewhat contradictory picture. Regional studies with smaller cohorts often suggest that maternal infection during early pregnancy may increase the risk or severity of NSOFC, which biologically aligns with the critical timing of craniofacial development in the first trimester. However, these signals lose strength when tested in larger population-based cohorts, where associations are weak, nonsignificant, or absent altogether. This raises the possibility that earlier findings may reflect sampling limitations, reporting bias, delays due to incomplete registries during the pandemic, or unmeasured confounders.

An emerging theme is the role of maternal stress and fear of COVID-19, which appear to exert measurable effects on pregnancy outcomes independent of infection status and, in some instances, show protective or paradoxical associations. This highlights the broader psychosocial dimension of the pandemic as a potential modifier of congenital anomaly risk. Taken together, the possibility of a modest or context-specific association between COVID-19 and CFAs cannot be entirely excluded. The consistency of large registry data suggests that infection alone is unlikely to be a major teratogenic factor. Instead, maternal psychosocial stressors, healthcare access, and timing of infection may be more relevant explanatory factors, emphasizing the need to consider both psychological and biological exposures.

Nationwide registries conducted in France, Scotland, the Netherlands, and Ontario, as well as the Sibling Matched Study and Nordic population studies, revealed that vaccination in the first trimester was associated with a lower risk of congenital anomalies, including CFAs [19,21-24]. It can be speculated that, in the absence of vaccination, outcomes could have differed, although no causal association can be concluded.

## Conclusions

The current evidence does not support a strong or consistent causal relationship between maternal COVID-19 infection and the occurrence of NSOFC. While smaller regional studies suggest a possible association, particularly with first-trimester exposure, these findings are not substantiated by larger population-based datasets. A plausible biological mechanism exists, as COVID-19 may indirectly influence craniofacial development through pathways such as mTOR dysregulation during early pregnancy, as suggested by pre-pandemic research; however, clinical evidence supporting this link remains inconsistent. This disparity underscores the influence of methodological limitations, reporting bias, and unmeasured confounders in earlier studies.

Emerging data also highlight the significant role of maternal psychosocial stress, healthcare access, and pandemic-related disruptions as potential modifiers of pregnancy outcomes. Reassuringly, evidence from multiple national registries supports the safety of COVID-19 vaccination in early pregnancy, with no increased risk of congenital anomalies. Overall, COVID-19 infection alone is unlikely to be a major teratogenic factor; instead, a multifactorial framework incorporating both biological and psychosocial determinants appears more relevant. Future research should focus on well-designed, large-scale studies with robust control of confounding variables to better elucidate these complex interactions.
